# Systematic and parasite-host relationship by *Baruscapillaria appendiculata* in *Phalacrocorax brasilianus* collected from Marajó Island, State of Pará, Brazil

**DOI:** 10.1590/S1984-29612023043

**Published:** 2023-07-21

**Authors:** Elaine Lopes de Carvalho, Ricardo Luis Sousa Santana, Evonnildo Costa Gonçalves, José Ledamir Sindeaux, Michele Velasco Oliveira da Silva, Elane Guerreiro Giese

**Affiliations:** 1 Laboratório de Histologia e Embriologia Animal, Instituto da Saúde e Produção Animal, Universidade Federal Rural da Amazônia - UFRA, Belém, PA, Brasil; 2 Programa de Pós-graduação em Saúde e Produção Animal na Amazônia, Instituto da Saúde e Produção Animal, Universidade Federal Rural da Amazônia - UFRA, Belém, PA, Brasil; 3 Laboratório de Tecnologia Biomolecular, Instituto de Ciências Biológicas, Universidade Federal do Pará - UFPA, Belém, PA, Brasil; 4 Universidade Federal Rural da Amazônia - UFRA, Belém, PA, Brasil

**Keywords:** Suliformes, Phalacrocoracidae, Capillariidae, Brazilian Amazon, Suliformes, Phalacrocoracidae, Capillariidae, Amazônia brasileira

## Abstract

The genus *Baruscapillaria* Moravec, 1982 has six valid species recorded in birds Phalacrocoracidae, namely *Baruscapillaria appendiculata* Freitas, 1933, *B*. *spiculata* Freitas, 1933, *B*. *carbonis* (Dubinin & Dubinina, 1940), *B*. *jaenschi* (Johnston & Mawson, 1945), *B*. *phalacrocoraxi* (Borgarenko, 1975) and *B*. *rudolphii* Moravec, Scholz and Našincová, 1994. Helminthological tests carried out on cormorants of the species *Phalacrocorax brasilianus* (Gmelin), a migratory bird that occurs in the northeast of the State of Pará, Brazil, demonstrate *B*. *appendiculata* parasitizing the cloaca of these birds, through light microscopy, scanning electron microscopy and molecular biology. These studies allowed a redescription of males and females of this nematode in these hosts and in this geographical area through integrative taxonomy. The occurrence of lesions in the cloaca caused by this nematode parasite was registered using histological analysis. This is a new geographic report for this nematode.

## Resumo

O gênero *Baruscapillaria* Moravec, 1982 possui seis espécies válidas registradas em aves Phalacrocoracidae, sendo *Baruscapillaria appendiculata* Freitas, 1933, *B*. *spiculata* Freitas, 1933, *B*. c*arbonis* (Dubinin & Dubinina, 1940), *B*. *jaenschi* (Johnston & Mawson, 1945), *B*. *phalacrocoraxi* (Borgarenko, 1975) e *B*. *rudolphii* Moravec, Scholz & Našincová, 1994. Exames helmintológicos realizados em mergulhões da espécie *Phalacrocorax brasilianus* (Gmelin), aves migratórias que ocorrem no nordeste do Estado do Pará, Brasil, demonstram *B*. *appendiculata* parasitando a cloaca dessas aves, através de microscopia de luz, microscopia eletrônica de varredura e biologia molecular. Estes estudos permitiram uma redescrição de machos e fêmeas deste nematódeo, neste hospedeiro e nesta área geográfica, através da taxonomia integrativa. Foi registrada a ocorrência de lesões na cloaca causadas pelo parasitismo desse nematódeo, por meio de análise histológica, sendo um novo registro geográfico elw.

## Introduction

The nematodes of the superfamily Trichinelloidea represent a large group with varied morphological and biological characteristics. Most species parasitize all vertebrate taxa, and affect various organs of the body (Moravec, 2001). Birds of the Phalacrocoracidae family have continental and coastal aquatic habits. They use these environments for reproduction and feeding and have a mainly piscivorous diet. There are few references in the literature regarding the fish species that constitute their diet (Piacentini et al., 2015; Oliveira et al., 2019).

Several studies on *Baruscapillaria* Moravec, 1982 of Phalacrocoracidae birds have been carried out and recorded. These include *Baruscapillaria carbonis* (Dubinin & Dubinina, 1940) in *Phalacrocorax carbo* (Linnaeus) in the Czech Republic, and in *Phalacrocorax brasilianus* (Gmelin) in Chile (Moravec et al., 1994; Frantová, 2001; González-Acuña et al., 2020), *B*. *jaenschi* (Johnston & Mawson, 1945) in *P*. *carbo*, *P*. *sulcirostris* (Brandt), *Microcarbo melanoleucos* (Vieillot), *P. fuscescens* (Vieillot) in Australia, *B*. *phalacrocoraxi* (Borgarenko, 1975) in *P*. *pygmeus* (Pallas) in the Asia (Baruš & Sergejeva, 1990b; Johnston & Mawson, 1945), *B. rudolphii* Moravec, Scholz & Našincová, 1994 in *P*. *carbo* in South Moravia and the Czech Republic (Moravec et al., 1994; Moravec & Scholz, 2016), *B. spiculata* (Freitas, 1933) Moravec, 1982 in *P*. *brasilianus* in Argentina (Garbin et al., 2021). And in Brazil, Monteiro et al. (2011) identified *B. appendiculata* Freitas, 1933 in *P*. *brasilianus*.

The goals of this study were therefore to report on *B. appendiculata* parasitizing *P. brasilianus* on Marajó Island, State of Pará, Brazil, and to provide an integrative taxonomic species redescription, bringing together the morphological and morphometric data, using optical and scanning electronic microscopy, and molecular analyses, using the partial 18S rDNA gene. Additionally, we present a histopathological analysis of lesions caused by this capillariid on the cloaca of this bird.

## Material and Methods

From 2020 to 2022, ten specimens of *P. brasilianus* were obtained from birds found trapped in fishing nets or trapped in fishing pens in the municipality of Soure (0° 13' 55” S; 48° 26' 58” W), Marajó Island, State of Pará, Brazil. The research has a license from ICMBio/SISBIO nº 74195 and license nº 6309230520 from the Ethics Committee in the use of animals. Only the organs of the digestive tract were sent frozen to the laboratory for a search for parasitic helminths. In the laboratory, the organs were separated and placed in Petri dishes with 0.9% NaCl saline solution and examined individually with the aid of a stereomicroscope (Leica ES2) in search of parasites. The taxonomic classification of nematodes was in accordance with Vicente et al. (1995), Moravec (1982), Moravec (2001), De Ley & Blaxter (2002) and Gibbons (2010). The ecological indices of parasitism were analyzed according to Bush et al. (1997), Bautista-Hernández et al. (2015) and Reiczigel et al. (2019).

The harvested nematodes were washed in 0.9% NaCl, fixed in AFA solution (93 parts of 70% ethyl alcohol, 5 parts of formaldehyde and 2 parts of glacial acetic acid) for 24 hours and then stored in 70% alcohol.

### Light microscopy

For light microscopy, nematodes were clarified in 0.5% Aman's Lactophenol solution and observed under a Leica DM2500 microscope with a drawing tube and photographed under a Leica DM2500 microscope with Leica camera system type DFC310 FX with Leica Application Suite Software V4 .4. and stored in glycerin alcohol (70% ethanol with 5% glycerin). Measurements are given in micrometers unless otherwise noted and are given as means followed by ranges in the parentheses.

### Scanning electron microscopy

For scanning electron microscopy (SEM), forty-five nematodes were fixed in 3% Glutaraldehyde and washed in 0.2M phosphate buffer solution. Each one was washed for one hour, then post-fixed in 1% Osmium Tetroxide, dehydrated in progressive alcohol for one hour each (50%, 70%, 80%, 90%, 100%), and dried at the CO_2_ critical point of, metallized with palladium-gold and observed in a TESCAN scanning electron microscope model VEGA 3 as per Carvalho et al. (2022).

### Molecular analysis

For molecular and phylogenetic analyses, 30 nematodes were used. The helminths were extracted from the cloaca and fixed in absolute alcohol. Total DNA was extracted with an Invisorb^®^ Spin Tissue Mini Kit (Invitek Molecular, Berlin, Germany), following the manufacturer's instructions. The SSU rDNA sequence was amplified with forward primers 18S-E (5'-CCGAATTCGTCGACAACCTGGTTGATCCTGCCAGT-3') and reverse primer 18S-A27 (3'- CCATACAAACGTCCCCGCCTG -5’) (Olson & Caira, 1999). The final polymerase chain reaction volume was 25 μL, containing 1 ng of DNA template, 20mM Tris pH 8.4, 50mM KCl, 2mM dNTP (Invitrogen^®^), 1mM Mg2Cl, 0.5 pmol of each primer and 0.2 units of Taq DNA polymerase (Invitrogen^®^). The amplification profile consisted of 5 min of initial denaturation at 95 °C, followed by 35 1 min cycles of at 94 °C, 1 min at 60 °C, and 1 min at 72 °C, followed by a final extension of 7 min. at 72 °C to polymerize any molecules that might have become dissociated from the polymerase prior to complete fragment synthesis.

The amplicons were submitted to electrophoresis in 1.5% agarose gel and purified with ExoSAP-ITTM (GE Healthcare, UK) and quantified using Nanodrop equipment (ThermoFisher, CA, USA). The samples were sequenced in the Applied Biosystems™ 3500 Genetic Analyzer (ThermoFisher, CA, USA), generating approximately 700 nucleotides for each sequence. The primers that were used to obtain the amplicons, were also used for sequencing.

The nucleotide sequences obtained from the samples were edited and aligned using the BioEdit software (Hall et al., 2011). After comparison with other sequences available in GenBank (BLAST search), the SSU rDNA sequence was aligned with the sequences of 18 species of capillariids available in GenBank. The database includes sequences from *Trichuris suis* (Schrank, 1788) and *Trichuris muris* (Schrank, 1788), which formed the outgroup for the phylogenetic analyses. The consensus sequence of nucleotides reported in the present study is available in GenBank databases.

Bayesian Inference (BI) and Maximum Likelihood (ML) was implemented using the Markov Chain Monte Carlo (MCMC) phylogenetic tree, implemented in MrBayes 3.1.2 (Ronquist & Huelsenbeck, 2003). This analysis was based on two parallel runs of four simultaneous MCMC searches of five million generations each, with one tree being sampled every 250 generations, after discarding the first 1000 trees. The remaining trees were analyzed with MrBayes to estimate the posterior probability of each node in the phylogenetic reconstruction. As indicated by jModelTest 2.1.9 (Darriba et al., 2012), the BI analysis assumed a TIM3ef + I + G model of nucleotide substitution, with the estimated base frequencies (A = 0.2573, C = 0.2202, G = 0.2821 and T = 0.2404), replacement model (A-C = 0.5953, A-G = 2.2582, A-T = 1.0000, C-G = 0.5953, C-T = 3.3969, G-T = 1.0000) and local variables after a gamma distribution (G = 0.5840), there were 88 models at the 100% confidence interval. Genetic distances were determined for the SSU rDNA sequences of capillariid species in PAUP 4.0 (Swofford, 1998).

### Histological processing

Three tissue fragments containing parasites inserted in the cloaca were fixed in 10% formaldehyde, dehydrated in increasing concentrations of 70%-100% ethanol, for 1 hour each and clarified in xylol in two baths, for 30 minutes each. Paraffin infiltration was performed with three successive baths in liquid paraffin for 20 minutes each in an oven at 60 °C followed by inclusion, after which they were sectioned into 5 μm thick sections using a ZEISS HYRAX M25 microtome (Tolosa et al., 2003). They were then stained with Hematoxylin-Eosin and Masson's Trichrome, and the images were obtained using a Leica DM 2500 microscope with a digital camera coupled to a LEICA type DFC310 FX with Leica Application Suite V4.4 software.

Voucher specimens were deposited in the Coleção de Invertebrados do Museu Paraense Emílio Goeldi (MPEG), Belém, Pará, Brazil: 5 males (MPEG.PLA 000389), (MPEG.PLA 000390), (MPEG.PLA 000391), (MPEG.PLA 000392), (MPEG.PLA 000393) and 5 females (MPEG.PLA 000394), (MPEG.PLA 000395), (MPEG.PLA 000396), (MPEG.PLA 000397), (MPEG.PLA 000398).

## Results

### Search data

A total of 142 nematodes were recovered from *P. brasilianus* with a prevalence of 80% (8 infected hosts out of 10 analyzed). This means a prevalence of 80%, mean intensity 17.75, mean abundance 14.2 and range of infection 1 to 45 nematodes per bird. All specimens collected showed characteristics compatible with *B. appendiculata* (Freitas, 1933) Moravec, 1982. The parasites were found embedded in the epithelium of the cloacal mucosa. Below are the results of the taxonomic identification of this nematode, performed using morphological, morphometric, molecular, and phylogenetic analyses, as well as analyses of the histopathology of its parasitism.

#### Nematoda

Enoplea Inglis, 1983

Trichinellida Hall, 1916

Capillariidae Railliet, 1915

*Baruscapillaria* Moravec, 1982

**Figure 1:**
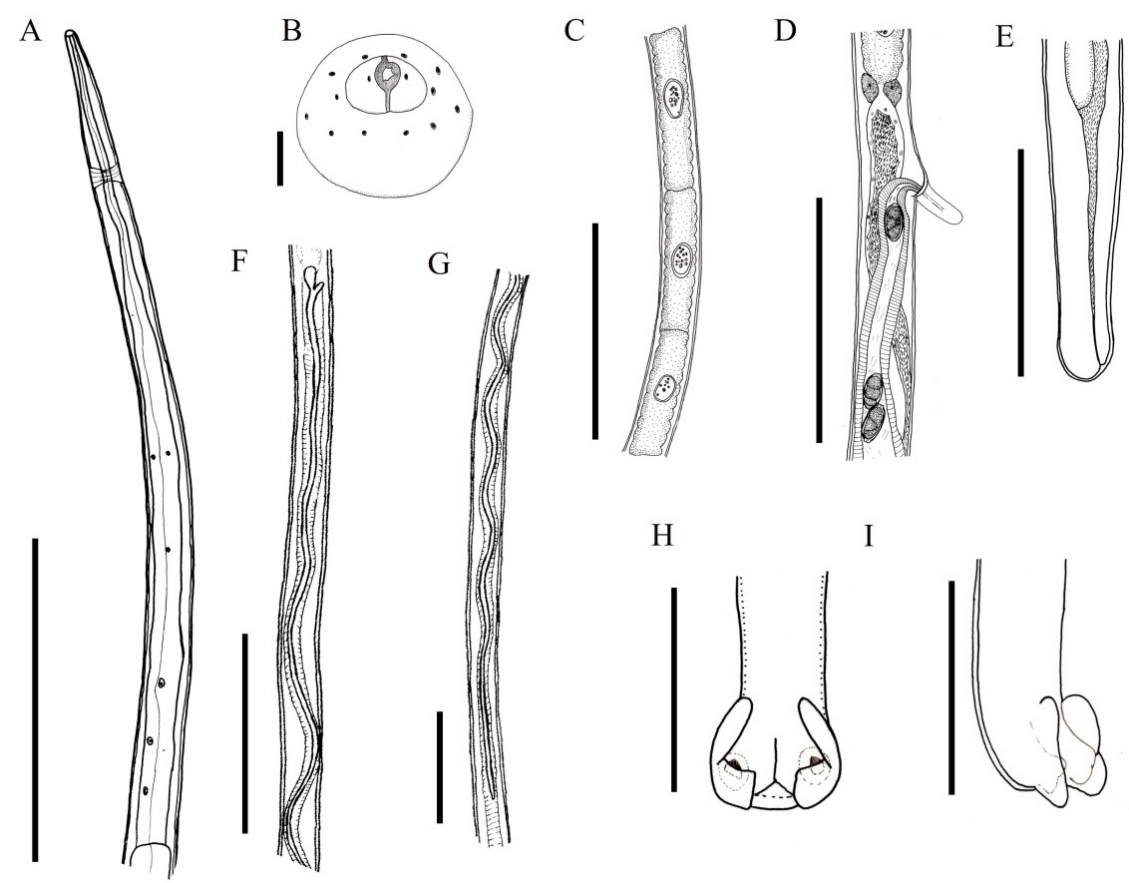
*Baruscapillaria appendiculata* from *Phalacrocorax brasilianus*. A. Anterior end of the female. Scale bar= 20µm. B. Cephalic region containing twelve pairs of cephalic papillae, one pair of amphids, and simple labia (reconstructed from SEM micrograph). Scale bar= 2µm. C. Stichocytes with large nuclei and numerous nucleoli. Scale bar= 30µm. D. Intestinal esophagus junction, shown vulva and vulvar appendix. Scale bar=30µm. E. Female's tail, lateral view. Scale bar= 20µm. F, G. Region of the base of the spicule and the tip of the spicule, respectively. Scale bar= 40µm. H. Male's tail ventral view. Scale bar= 10µm. I. Male's tail, lateral view. Scale bar=10µm.

*Baruscapillaria appendiculata* (Freitas, 1933) Moravec, 1982

(Description based on light microscopy and scanning electron microscopy: Figures 1 [Fig gf03] [Fig gf04]-5)

**Figure 3 gf03:**
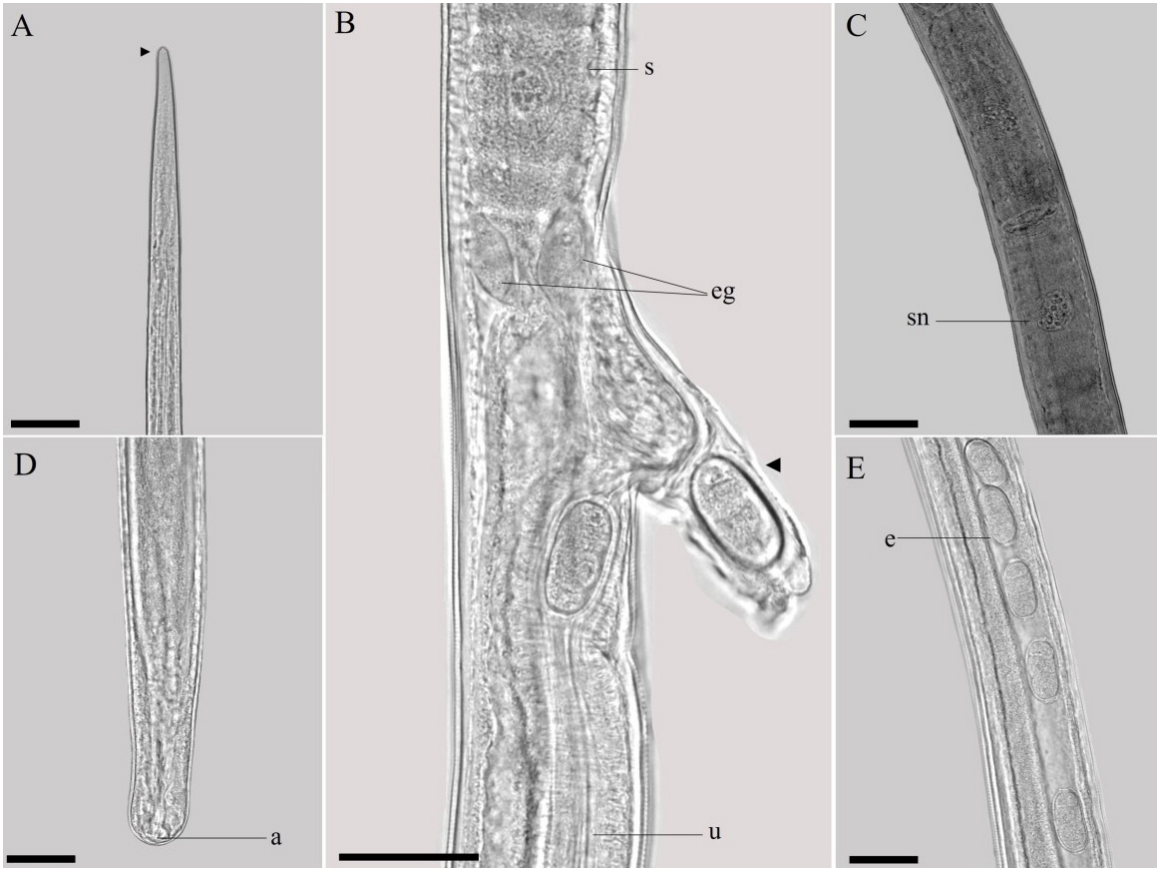
Light microscopy of female *Baruscapillaria appendiculata*. A. Anterior extremity, cephalic region (black arrowhead). Scale bar= 50µm. B. Intestinal esophagus junction, note stichocytes (s), esophageal glands (eg), vulva with vulvar appendage (black arrowhead) containing an egg and uterus (u). Scale bar= 100µm. C. Stichocytes with large nucleus and nucleoli inside (sn). Scale bar= 50µm. D. Posterior end, anus (a). Scale bar= 50µm. E. Embryonated eggs (e) in utero. Scale bar= 50µm.

**Figure 4 gf04:**
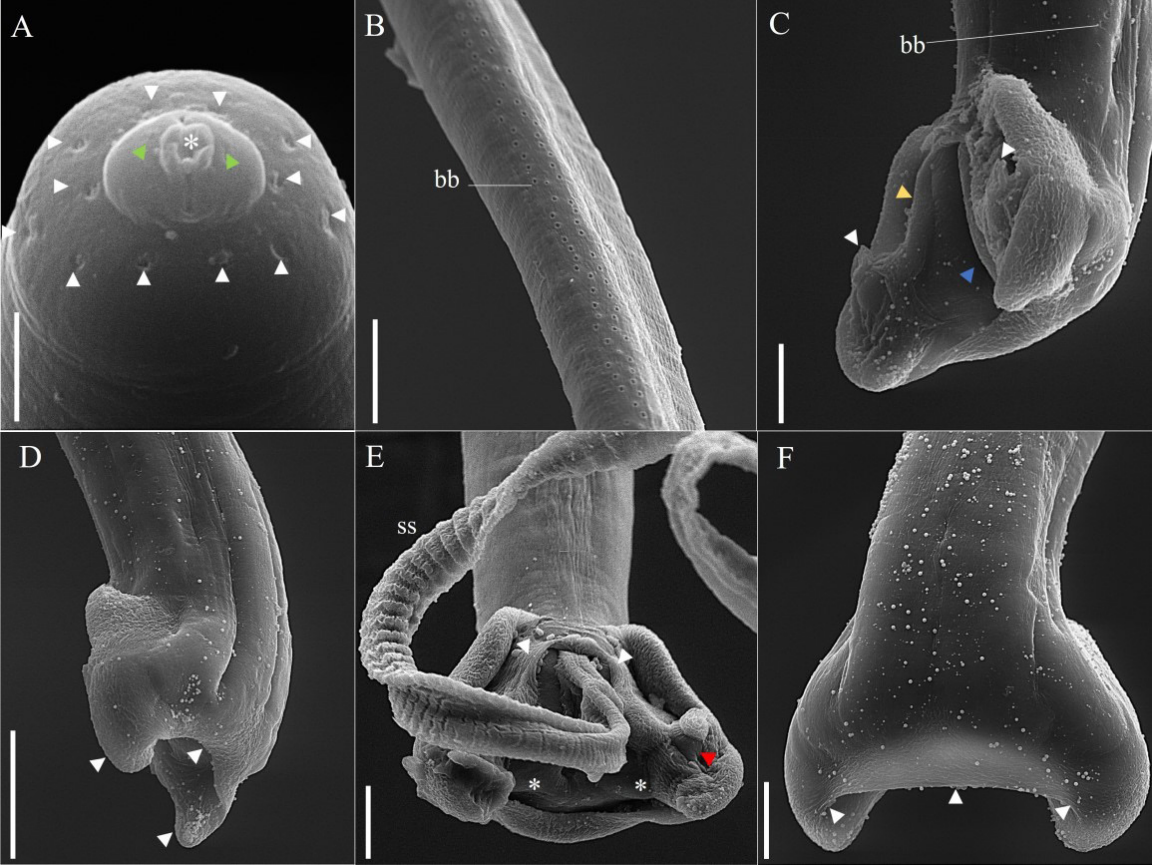
Scanning electron microscopy of male *Baruscapillaria appendiculata* from *Phalacrocorax brasilianus*. A. Button-shaped anterior end, simple lips (*), presence of amphids (green arrowhead) and twelve pairs of cephalic papillae (white arrowhead). Scale bar= 2µm. B. Lateral bacillary band (bb). Scale bar= 20µm. C. Tail, ventrolateral view, observe the bacillary band (bb), papillae (white arrowhead) in each lobe and membrane surrounding the cloaca (yellow arrow) and ventral/medial face of the caudal lobes interconnecting the papillae, cloaca (blue arrowhead). Scale bar= 20µm. D. Posterior extremity, lateral view, membranous bursa (white arrowhead) is observed. Scale bar= 20µm. E. Tail, ventral view, with transverse striations spicular sheath (ss) exposed caudal lobes (*) containing one large papilla each (red arrowhead) where these papillae have a membrane surrounding the cloaca and lobes (white arrowhead). Scale bar= 10µm. F. Dorsal view of the membranous bursa (white arrowhead). Scale bar= 10µm.

**Figure 5:**
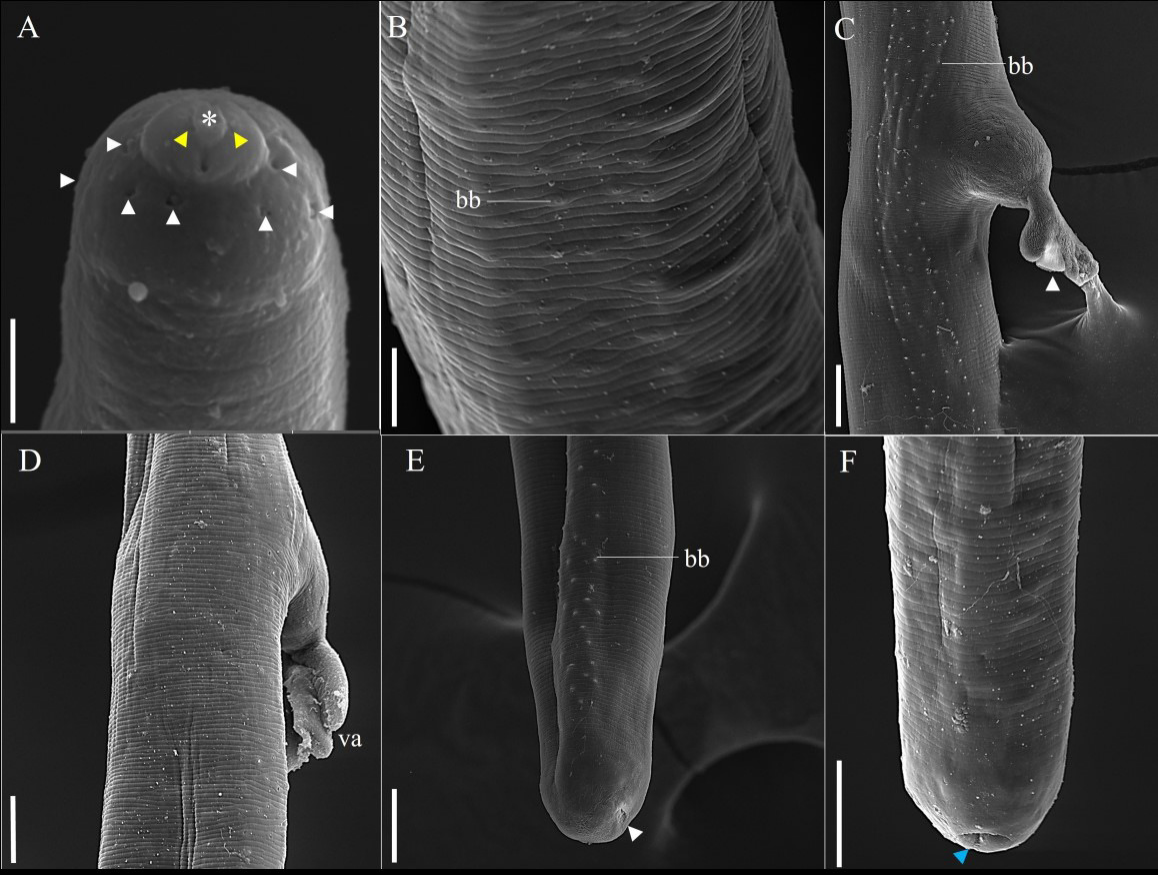
Scanning electron microscopy of female *Baruscapillaria appendiculata* of *Phalacrocorax brasilianus*. A. Button-shaped anterior end, simple lips (*), presence of amphids (yellow arrowhead) and cephalic papillae (white arrowhead). Scale bar= 10µm. B. Lateral bacillary band (bb). Scale bar= 10µm. C. Lateral view of the female's bacillary band (bb), vulva region and well-developed vulvar appendix (white arrowhead). Scale bar= 20µm. D. Dorsal view of the female showing the ventral surface of the vulvar appendix (va). Scale bar: 20µm. E. Lateral view of the female's tail, bacillary band (bb) and anal opening (white arrowhead). Scale bar: 20µm. F. Ventral view of the tail, note the anal opening (blue arrowhead). Scale bar= 20µm.

Long-bodied, threadlike nematodes with transversely striated cuticle. Anterior region containing twelve papillae and a pair of amphids. Oral opening circular in shape. Short, narrow muscular esophagus. Nerve ring located in the initial portion of the muscular esophagus. Stichosome consisting of a single row of 43 elongated stichocytes with distinct transverse rings; markedly large stichocytes nuclei and many nucleoli. Two pseudocoelomate glandular cells present at the esophagus-intestine junction. Two bacillary lateral bands along the body in both males and females.

Male (based on 10 specimens with exposed sheath): Body length 14 mm (11-16); and maximum width at the junction between the esophagus and intestine of 48 (40-70). Length of muscular esophagus 323 (267-370) × 14 (13-17), of stichosome 4.82 mm (1.97-6.34), number of stichocytes about 9 (7-14), stichocytes with distinct transverse rings; large stichocytes nuclei. Length of entire esophagus 5.14 mm (4.90-6.84), representing 39% of body length. Nerve ring situated 66 (43-80) from anterior end. Spicule single, sclerotized, measuring 2.07 mm (1.96-2.29) × 10 (8-12); proximal end of spicule rounded. Aspinous spicular sheath, transverse striations, widely spaced and almost smooth in some regions. Posterior end of body truncated, with two distinct, rounded ventrolateral lobes and a pair of large papillae containing a membrane on each papilla. Terminal cloacal opening. Membranous bursa present.

Female (based on 10 gravid specimens): Body length 25 mm (21-29); and maximum width at the junction between the esophagus and intestine of 62 (53-82). Length of muscular esophagus 452 (348-523) × 18 (17-23), of stichosome 5.68 mm (4.68-7.21), number of stichocytes about 43 (39-46), stichocytes with distinct number of transverse rings; large stichocytes nuclei. Length of entire esophagus 6.15 mm (5.17-7.44), representing 24% of body length. Nerve ring situated 87 (67-130) from anterior end. Distance from the end of the stichocytes to the vulva 130 (58-233). Long vulvar appendage 64 (50-78). Eggs arranged in a single row near the exit of the vagina. Barrel-shaped eggs 43 (42-47) × 21 (20-23), with slightly protruding polar plugs. Egg wall with thick hyaline layer, thin superficial crenate outer layer. Caudal end rounded, anus subterminal.

### Molecular characterization and phylogenetic analysis

The rDNA gene sequence obtained for *B*. *appendiculata* was 1744 bp long and is available on GenBank (accession nº OP828910). A BLAST search revealed that the nucleotide sequences with the greatest similarity were those of *B*. *spiculata* (accession nº MT068209) described in a grebe from Argentina with 98.83% similarity, and *Aonchotheca putorii* (Rudolphi, 1819) (accession nº MT127177) in a mammal from Japan, with 97.32% similarity.

Molecular characterizations available for *Baruscapillaria* show the type species *B*. *obsignata* (Madsen, 1945), and the species *B*. *spiculata* in which the 18S rDNA region was amplified. Therefore, we performed a phylogenetic study to confirm the taxonomic status and generic attribution of our research species. The isolate in the present study showed 98.83% identity with *B*. *spiculata*. The pairwise genetic distance between the isolates was 0.010 (Table 1). Consequently, these specimens can be considered to belong to the same genus, *Baruscapillaria*.

**Table 1:** Pairwise genetic distance data (p-distance) between known capillariid species.

	(1)	(2)	(3)	(4)	(5)	(6)	(7)	(8)	(9)	(10)	(11)	(12)	(13)	(14)	(15)	(16)	(17)	(18)	(19)
(1) *Baruscapillaria appendiculata*	-																		
(2) *B*. *spiculata* MT068209	0.010																		
(3) *B*. *obsignata* LC425004	0.039	0.044																	
(4) *Aonchotheca paranalis* MF621021	0.058	0.067	0.043																
(5) *Aonchotheca* sp. LC052374	0.067	0.072	0.045	0.024															
(6) *A. putorii* LC052356	0.063	0.070	0.040	0.021	0.016														
(7) *A. musimon* LC052379	0.068	0.076	0.046	0.029	0.022	0.018													
(8) *Eucoleus* sp. LC052381	0.161	0.165	0.133	0.133	0.113	0.115	0.120												
(9) *Eucoleus* sp. LC052382	0.162	0.169	0.140	0.128	0.117	0.118	0.125	0.030											
(10) *E. contortus* LC424996	0.171	0.176	0.145	0.152	0.134	0.132	0.143	0.046	0.047										
(11) *E. dispar* EU004821	0.175	0.178	0.155	0.159	0.145	0.143	0.149	0.049	0.057	0.023									
(12) *E. aerophilus* MW709573	0.188	0.196	0.161	0.163	0.146	0.144	0.152	0.048	0.048	0.020	0.019								
(13) *E. garfiai* LC484432	0.158	0.156	0.137	0.143	0.129	0.128	0.141	0.038	0.039	0.016	0.013	0.008							
(14) *E. perforans* LC424997	0.180	0.187	0.155	0.158	0.140	0.141	0.145	0.049	0.052	0.032	0.034	0.031	0.027						
(15) *Capillaria madseni* LC052347	0.170	0.171	0.157	0.152	0.146	0.154	0.154	0.196	0.201	0.198	0.208	0.216	0.185	0.209					
(16) *C. anatis* LC425001	0.169	0.170	0.145	0.149	0.143	0.144	0.152	0.180	0.190	0.187	0.198	0.196	0.171	0.196	0.096				
(17) *C. pudendotecta* LC052339	0.216	0.219	0.185	0.190	0.169	0.181	0.187	0.213	0.222	0.215	0.237	0.228	0.209	0.241	0.144	0.113			
(18) *C. spinulosa* LC424999	0.220	0.225	0.197	0.193	0.175	0.189	0.192	0.226	0.233	0.227	0.249	0.238	0.221	0.252	0.146	0.133	0.028		
(19) *C. tenuissima* EU004822	0.234	0.244	0.203	0.207	0.197	0.203	0.208	0.242	0.240	0.225	0.239	0.248	0.222	0.249	0.144	0.157	0.185	0.196	-

**Figure 6:**
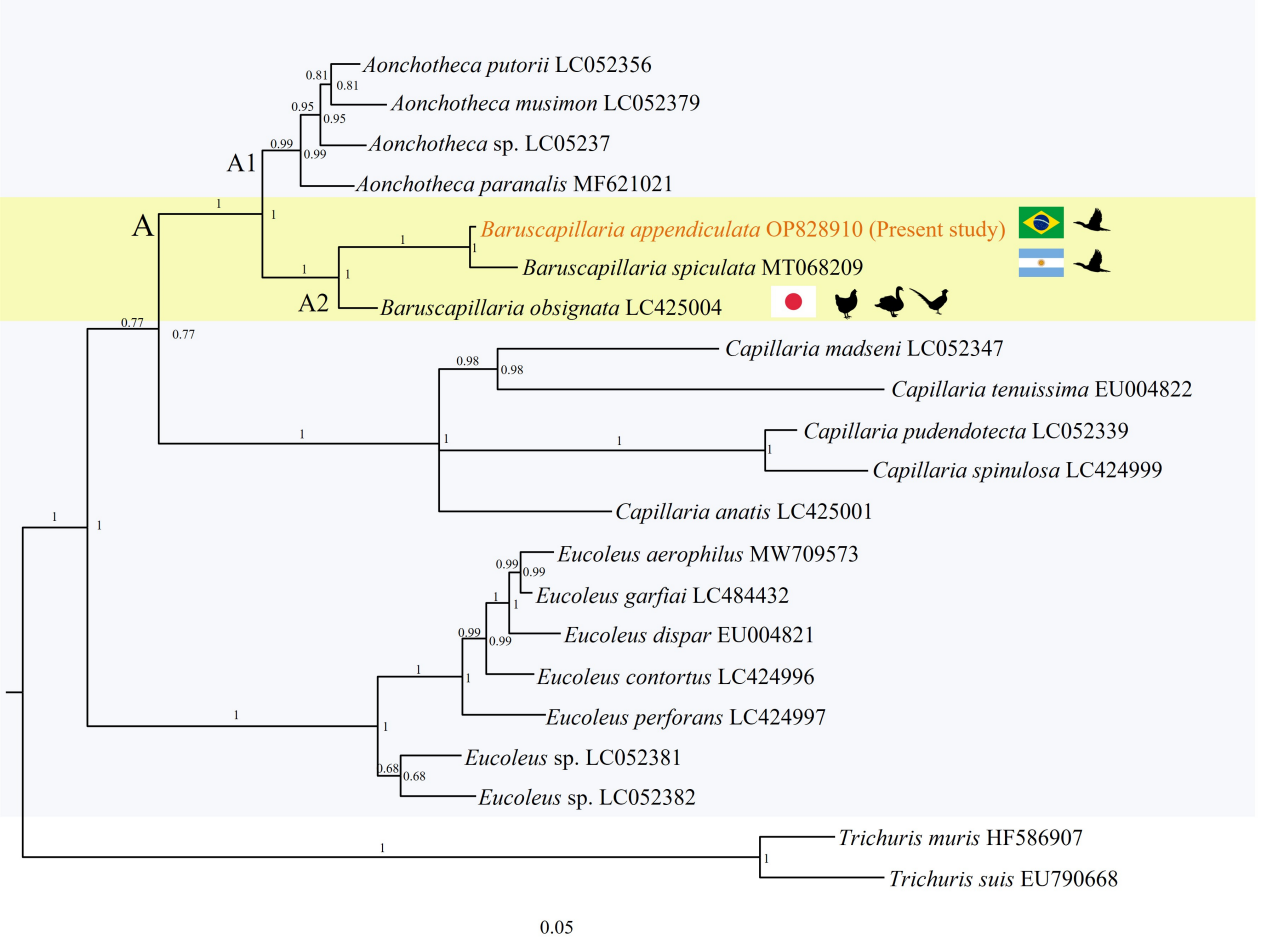
Bayesian phylogenetic tree based on the 18S rDNA sequence obtained from the SSU rDNA analysis of *Baruscapillaria appendiculata* compared to other capillariids. Node numbers represent posterior probability values calculated from BI/bootstrap ML values (> 50%). The scale bar indicates the number of mutations per sequence position. Data are displayed with names of species.

Phylogenetic analysis based on 18 18S rDNA sequences from Capillariidae species was performed by BI and ML producing well-resolved topologies (Figure 6). Paired DNA analyzes showed genetic distances between the studied taxa ranging from 0.010 to 0.252. The values between *B. appendiculata* (Present study) and *B*. *spiculata* MT068209 (0.010) were the lowest observed (Table 1).

The isolate from present study formed a well-supported clade A with isolates of *A. putorii, A. musimon*, *A*. sp., *A. paranalis* forming the A1 subclade, which are parasites of mammals in Japan and Poland. *B*. *obsignata* and *B*. *spiculata,* which are parasites of chickens, pheasants, and ducks in Kagoshima and Yamaguchi in Japan, and cormorants in Argentina formed A2 subclade (Tamaru et al., 2015; Garbin et al., 2021). In the A2 subclade *B*. *appendiculata* of the present study formed a sister clade with *B*. *spiculata* having 0.010 of genetic distance (Table 1), although both present a host in common. *B*. *appendiculata* has morphological characters in the spicular sheath that distinguish it from *B*. *spiculata*. Still in this subclade *B*. *obsignata* has 3.9% of genetic distance in relation to present study, although the only similarity is that they all belong to the same genus.

### Histological analysis

**Figure 7:**
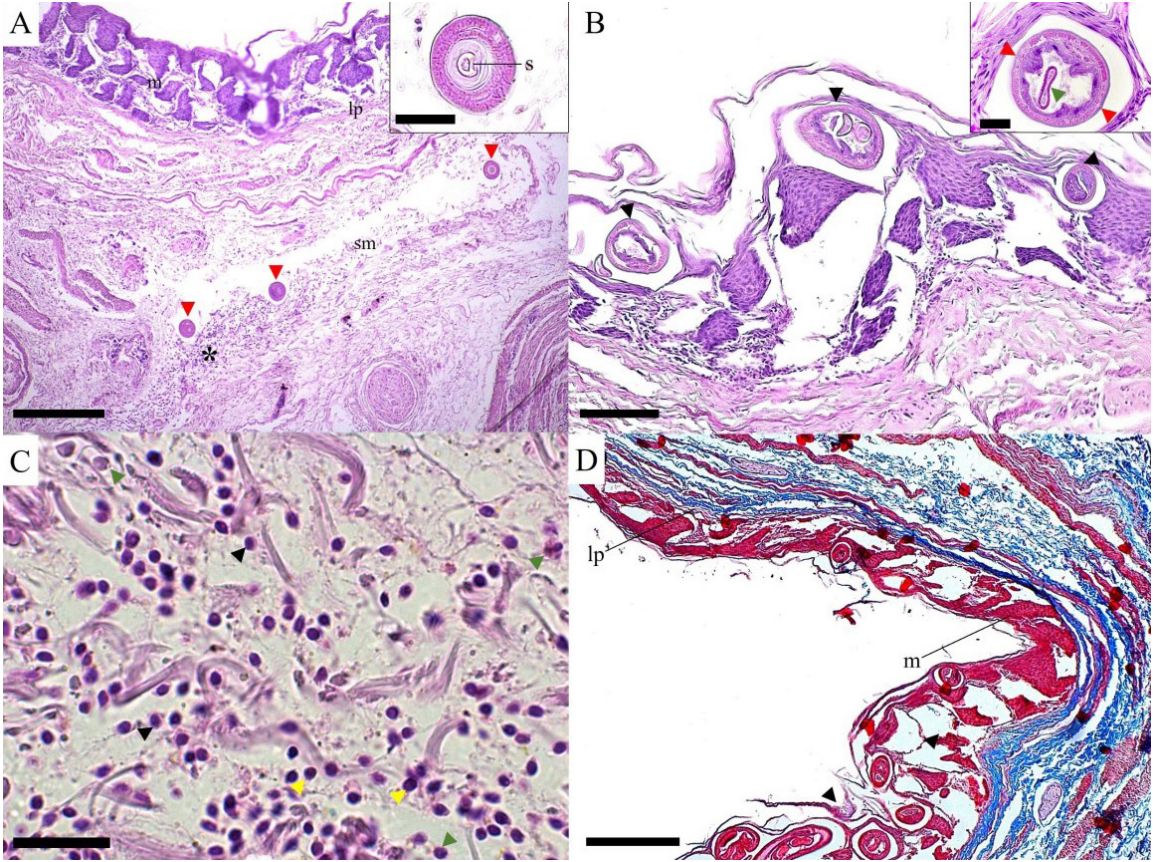
Photomicrograph of histological section of the cloaca of *Phalacrocorax brasilianus*. A. Cross section of the cloaca showing the mucous layer (m), lamina propria (lp) and submucosal layer (sm) with the presence of three cross sections of a *Baruscapillaria appendiculata* male (red arrowhead) and area with inflammatory infiltrate (*) in the submucosal layer (sm). Scale bar = 200µm. In the insert is observed the male's tail where is observed a cross section of the spicule (s). Scale bar = 25µm. Hematoxylin-eosin staining. B. In the mucous layer, is observed cross-sections of female *B*. *appendiculata* (black arrowhead). Scale bar = 100µm. In the insert there is a cross-section of the female with is observed bacillary (red arrowhead) and egg (green arrowhead) bands. Scale bar = 20µm. Hematoxylin-eosin staining. C. Area of inflammatory infiltrate, lymphocytes (yellow arrowhead), eosinophils (green arrowhead) and plasma cells (black arrowhead). Scale bar = 20µm. Hematoxylin-eosin staining. D. Mucous layer (m) with pregnant females of *B*. *appendiculata* causing great destruction of the layer (black arrowhead), without affecting the lamina propria (lp). Scale bar = 200µm. Masson's trichrome stain.

In the fresh tissue samples examined by light microscopy it was possible to observe numerous parasites in the cloaca mucosa and hyperemic areas resulting from this parasitism. In the histological section, males, and pregnant females of *B*. *appendiculata* were shown with plasmocytes, some lymphocytes and eosinophils, characterizing a moderate inflammatory infiltrate. We observed that the lesion is predominant in the mucous layer where the females are inserted in their tunnels and can transpose the muscularis mucosa and affect the submucosa (Figure 7).

## Discussion

According to Moravec (1982), the genus *Baruscapillaria* is diagnosed as having well-developed membranous bursa supported on both sides by one or two small, rounded lobes narrowed at the base; each lobe has a minute projection, usually ventrally folded and a long, well-sclerotized spicule, with a non-spiny spicular sheath. They are parasites of the intestine and stomach of birds and mammals. Freitas (1933a, b) in the original description of *B*. *appendiculata* described males as having a posterior end provided with two lobes in the form of an “L” involved in a rudimentary caudal bursa and smooth spicular sheath, and as parasites in the large intestine of *P*. *brasilianus* in Rio de Janeiro. In the description of *B*. *spiculata*, the male presented a posterior end with four papillae on the tail and the sheath presents spiral striation, distinct throughout most of its extension and parasitizing the cloaca of the same host. In our study, the morphological analysis made it possible to report *B*. *appendiculata* in *P*. *brasilianus*, in which the characteristics of the sheath were of major importance for comparison with the species described by Freitas (1933a, b).

Moravec (1982) proposed a new systematic arrangement in the Capillariidae family, reclassifying (according to morphological characters) *Capillaria appendiculata*, originally described by Freitas (1933a), to *B*. *appendiculata*. Baruš & Sergejeva (1990a) registered a new genus called *Ornithocapillaria*, including only species that parasitized the intestine of birds of the orders Passeriformes, Falconiformes, Strigiformes, and Piciformes. Moravec et al. (2000) used the generic epithet *Ornithocapillaria* to describe specimens found in fish as *O*. *appendiculata*. Later in his book, Moravec (2001) listed *O*. *appendiculata* as a synonym of *B*. *appendiculata*, the most current classification. However, we still find divergences regarding the nomenclature used in different studies (Monteiro et al., 2011; Garbin et al., 2021). The present study corroborates the identification of *B*. *appendiculata* parasitizing *P*. *brasilianus*, with the cloaca being the site of infection.

**Figure 2:**
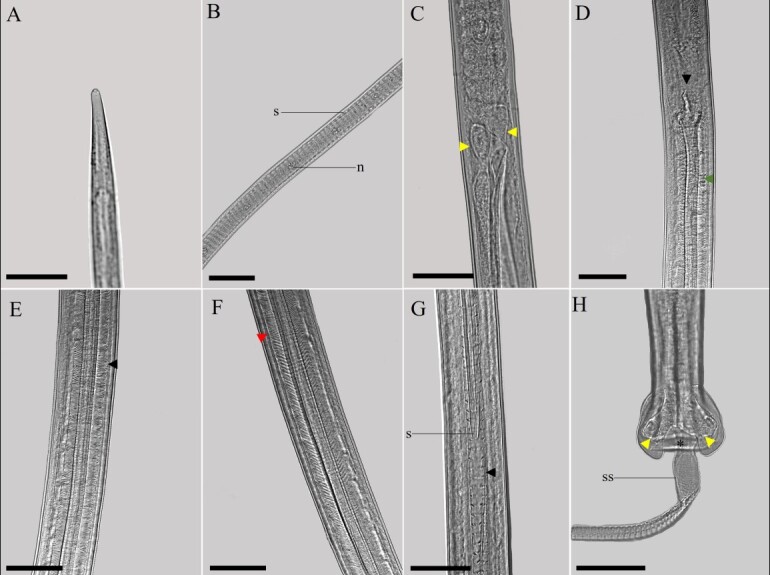
Light microscopy of male *Baruscapillaria appendiculata*. A. Anterior extremity, cephalic region. Scale bar= 50µm. B. Anterior end, note stichocytes (s) and stichocytes nucleus (n). Scale bar= 100µm. C. Intestinal esophagus junction, note the esophageal glands (yellow arrowhead). Scale bar= 50µm. D. Posterior end of male, base of spicule (black arrowhead) and beginning of spicular sheath (green arrowhead) showing transverse striations. Scale bar= 50µm. E. Spicular sheath containing transverse striations, more compact transverse striations can be seen in the proximal section (black arrowhead). Scale bar= 50µm. F. Second cut with transverse striations becoming looser (red arrowhead). Scale bar= 50µm. G. Note tip of spicule (s) and distal segment with transverse striations becoming wider and looser when the spicular sheath is extruded (black arrowhead). Scale bar= 50µm. H. Posterior end, the male's tail can be seen with slightly lateralized caudal lobes (yellow arrowhead) containing a membranous bursa (*) and exposed spicular sheath (ss). Scale bar=50µm.

In the most recent study, Garbin et al. (2021) redescribed *B*. *spiculata* in *P*. *brasilianus* from Argentina, with a spicular sheath marked by four distinct regularly patterned sections, subterminal cloacal opening and caudal end with a well-developed membranous bursa. In the present study was observed many morphological similarities with what was described by the authors above, such as the shape of the caudal end of the male. However, when comparing *B*. *spiculata* and *B*. *appendiculata* in the present study significant differences regarding the spicular sheath (Figure 2D-2H). The specimens of *B*. *appendiculata* deposited by Freitas in 1933 (Freitas, 1933a) at CHIOC were not available for consultation, and those deposited by other researchers from the same period were very damaged, making visualization impossible.

Specimens of *B*. *appendiculata* capillariids were recorded by Moravec et al. (2000) in *Chirostoma estor* Jordan, 1880 and *Cyprinus carpio* Linnaeus, 1758; however, the authors report that the occurrence in these fish suggests that these nematodes may have been accidentally acquired while the fish were feeding on grebe droppings containing nematodes. As a result, Moravec (2001) in his work rectified *C*. *estor* and *C*. *carpio* as accidental hosts of *B*. *appendiculata* found in the intestine of these fish. This was confirmed in the present study, where we recorded the adult forms in the cloaca of birds, and with pregnant females in all collections of *P*. *brasilianus*.

Monteiro (2006) recorded *B*. *appendiculata* parasitizing the large intestine and cloaca of *P*. *brasilianus* in southern Brazil; however, in the description of these capillariids they observed a non-spiny spicular sheath in males with three distinct regions (reticulate, stellate, and helical), and presence of four bacillary bands, in both. Nonetheless, Garbin et al. (2021) analyzed the specimens described by Monteiro (2006) and concluded that it could be *B*. *spiculata* and not *B*. *appendiculata*. In the present study, the specimens were morphologically identified as *B*. *appendiculata*, which has a reticulate spicular sheath and, as the sheath expands, the reticulate shape becomes more discrete, resembling a smooth sheath, which differs from *B*. *spiculata* by not have four distinct sections in the spicular sheath, giving a spiral shape as originally described by Freitas (1933b) and reaffirmed by Garbin et al. (2021). The comparison of the morphological and morphometric data of the present study with previously published *Baruscapillaria* species is shown in Table 2.

**Table 2:** Morphology and morphometric data comparison of *Baruscapillaria appendiculata* from *Phalacrocorax brasilianus* in State of Pará, Brazil, collected in the present study with *Baruscapillaria* species previously published.

Morphometric characterization	*Baruscapillaria appendiculata*		*B. obsignata*		*B. spiculata*		*B. appendiculata*		*B. obsignata*		*B. obsignata*		*B. jaenschi*		*B. phalacrocoraxi*		*B. appendiculata*
Specimen sex	Male	Female	Male	Female	Male	Female	Male	Female	Male	Female	Male	Female	Male	Female	Male	Female	Female
Host	*Phalacrocorax brasilianus*		chicken and turkey		*P. brasilianus*		*P. brasilianus*		*Cygnus atratus*		Chicken		*P. sulcirostris, P. fuscescens. P. carbo, P. melanoleucas*		*P. pygmeus*		*Cyprinus carpio*
			Small intestine		Cloaca		Large intestine		Small intestine		Small intestine		?		Bursa Fabricius, cloaca		Intestine
Locality	Pará, Brazil		USA		Rio de Janeiro, Brazil		Rio de Janeiro, Brazil		UK		UK		Australia		Asia		Lake Pátzcuaro, Mexico
Total body (L)^a^	**11-16**	**21-29**	8.60-10	10-12.70	16	28	-	22.8	12.35-14.79	16.90-20.50	6.90-12.96	8.28-17.28	9.9	7.1-27.4	9.1-13.7	19.8-24.3	21.83
Maximum body (W) b^,^^c^	**40-70**	**53-82**	53	-	70	100	64	88-96	46-49	56-64	42-51	49-67	36	37-41	55-66	88-90	75
Nerve-ring (L) ^c,^^d^	**43-80**	**67-130**	-	-	-	-	-	-	-	-	-	-	-	-	-	-	111
Muscular esophagus (L) ^c^	**267-370**	**348-523**	-	-	-	-	-		-	-	-	-	-	-	-	-	399
Muscular esophagus (W) ^c^	**13-17**	**17-23**	-	-	-	-	-	-	-	-	-	-	-	-	-	77	-
Total esophagus (W) ^a^	**4.90-6.84**	**5.17-7.44**	-	-	6-7	6-7	-	6.3	-	-	4.23-6.00	4.94-6.70	-	-	3.9-4.3	5.6-5.9	4.78
Stichosome (W) ^a^	**1.97-6.34**	**4.68-7.21**	-	-	-	-	-	-	-	-	-	-	-	-	-	-	4.39
# Stichocytes	**41-52**	**39-46**	-	-	-	-	-	-	-	-	-	-	-	-	-	-	39
Vulva (L) ^a^	**-**	**0.058-0.233**^e^	-	-	-	0.130 ^e^	-	0.072 ^e^	-	-	-	-	-	-	-	0.1^e^	0.081^e^
Eggs mature (L×W) ^c^	**-**	**43.27×21.52**	-	50-62	-	40-36	-	56-32	-	42-51×22-27	-	44-56×22-29	-	48-50×18-20	-	44-45×22-23	-
Spicule (L) ^a^	**1.96-2.29**	**-**	1.20	-	2.33	-	1.77	-	1.59-1.78	-	1.09-1.53	-	7	-	3.08-3.30	-	-
Spicule (W) ^d^	**8-12**	**-**	-	-	-	-	-	-	-	-	-	-	-	-	22-25	-	-
ME/BL (%)	**2.41**	**1.78**	-	-	-	-	-	-	-	-	-	-	-	-	-	-	-
TE/BL (%)	**39**	**24.30**	-	-	14.5	-	-	27.6	-	-	-	-	-	-	-	-	-
# Specimen	**10**	**10**	2	3	2	2	2 fragmented	not specified	7	8	100	100	1	?	-	-	1
Reference	**In this study**		Graybill, 1924		Freitas, 1933b		Freitas, 1933a		Wakelin, 1963		Wakelin, 1965		Johnston & Mawson, 1945		Baruš & Sergejeva, 1990a		Moravec et al., 2000
Morphometric characterization	***Baruscapillaria appendiculata***		*B. appendiculata*		*B. appendiculata* ^h^		*B. obsignata*		*B. obsignata*		*B. obsignata*						
Specimen sex	**Male**	**Female**	Male	Female	Male	Female	Male	Female	Male	Female	Male	Female					
Host	***Phalacrocorax brasilianus***		*Chirostoma estor*		*P. brasilianus*		Chicken		*Cygnus olor*		Pheasant						
Site of infection	**Cloaca**		Intestine		Cloaca/large intestine		Small intestine		Small intestine		Small intestine						
Locality	**Pará, Brazil**		Lake Pátzcuaro, Mexico		Rio Grande do Sul, Brazil		Kagoshima, Japan		Yamaguchi, Japan		Kumamoto, Japan						
Total body (L)^a^	**11**-**16**	**21-29**	9.25^f^	14.88-22.13	12.90-16.10	20.70-25.90	5.31-10.61	6.19-10.56	8.58-10.38	10.36-13.67	7.61-8.60	9.02-16.84					
Maximum body (W) ^b, c^	**40**-**70**	**53-82**	48	60-81	42-60	65-90	28-56	52-64	40-58	57-80	35-39	52-65					
Nerve-ring (L) ^c, d^	**43-80**	**67-130**	-	84-153	70-90	-	-	-	-	-	-	-					
Muscular esophagus (L) ^c^	**267-370**	**348-523**	-	315-435	360-477	410-530	-	-	-	-	-	-					
Muscular esophagus (W) ^c^	**13-17**	**17-23**	-	-	-	-	-	-	-	-	-	-					
Total esophagus (W) ^a^	**4.90-6.84**	**5.17-7.44**	-	4.54-6.11	4.60-5.70	4.90-7.90	3.61-6.61	3.94-5.64	4.41-5.42	4.66-5.92	4.10-4.97	4.53-5.71					
Stichosome (W) ^a^	**1.97-6.34**	**4.68-7.21**	-	4.21-5.79	4.60-5.70	4.40-7.50	-	-	-	-	-	-					
# Stichocytes	**41-52**	**39-46**	-	40-45	45-49	43-45	-	-	-	-	-	-					
Vulva (L) ^a, f^	-	**0.058-0.233^e^**	-	0.093-0.171^e^	-	0.057-0.137^e^	-	0.039-0.072^e^	-	0.027-0.110 ^e^	-	0.035-0.076^e^					
Eggs mature (L×W) ^c^	-	**43.27×21.52**	-	57-60×27	-	49-23	-	48-59×24-31	-	-	-	48-26					
Spicule (L) ^a^	**1.96-2.29**	-	2.31	-	2.00-3.90	-	0.87-1.26	-	1.03-1.59	-	1.05-1.18	-					
Spicule (W) ^c^	**8**-**12**	-	0.012	-	17-30	-	-	-	-	-	-	-					
ME/BL (%)	**2.41**	**1.78**	-	-	2.50-3.10	2.90-4.90	-	-	-	-	-	-					
TE/BL (%)	**39**	**24.30**	-	22-41^g^	36.05	27.38	-	-	-	-	-	-					
# Specimen	**10**	**10**	1	6	8	6	22	7	9	6	4	3					
Reference	**In this study**		Moravec et al., 2000		Monteiro, 2006		Tamaru et al., 2015				Sakaguchi et al., 2020						
Morphometric characterization	***Baruscapillaria appendiculata***		*B. obsignata*		*B. obsignata*		*B. obsignata*		*B. spiculata*		*B. spiculata*		*B. kamanae*				
Specimen sex	**Male**	Male	Male	Male	Male	Female	Male	Female	Male	Female	Male	Female	Male	Female			
Host	***Phalacrocorax brasilianus***		Pigeon		Goose		Turkey		*P. brasilianus*		*P. brasilianus*		*Podiceps cristatus australis*				
Site of infection	**Cloaca**		Small intestine		Small intestine		Small intestine		Cloaca		Cloaca		Intestine				
Locality	**Pará, Brazil**		Surabaya, Indonesia		Surabaya, Indonesia		Surabaya, Indonesia		Argentina i		Argentina j		New Zealand				
Total body (L)^a^	**11**-**16**	**21-29**	8.58-11.00	12.67-16.28	7.23-9.67	8.00-12.30	8.95-10.50	12.69-14.65	10.75-17.60	24.00-28.18	12.55-17.25	23.5-27.8	15.2**-**15.8	23.9**-**24.3			
Maximum body (W) ^b, c^	**40**-**70**	**53-82**	40-59	66-76	41-45	49-72	41-67	53-75	50-60	60-80	46-60	55-78	51-55	78**-**86			
Nerve-ring (L) ^c, d^	**43-80**	**67-130**	-	-	-	-	-	-	30-75	55-105	35-65	45-95	**-**	**-**			
Muscular esophagus (L) ^c^	**267-370**	**348-523**	-	-	-	-	-	-	255-560	435-670	280-580	390-630	**-**	**-**			
Muscular esophagus (W) ^c^	**13-17**	**17-23**	-	-	-	-	-	-	-	-	-	-	**-**	**-**			
Total esophagus (W) ^a^	**4.90-6.84**	**5.17-7.44**	3.96-5.24	5.22-6.95	4.48-5.15	4.21-6.20	4.65-5.27	5.19-6.31	4.25-5.95	6.35-7.98	3.85-6.05	5.80-7.50	8.4	7.5**-**10.3			
Stichosome (W) ^a^	**1.97-6.34**	**4.68-7.21**	-	-	-	-	-	-	3.85-5.64	5.90-7.12	4.27-5.61	6.10-7.32	**-**	**-**			
# Stichocytes	**41-52**	**39-46**	-	-	-	-	-	-	32-46	37-48	34-44	35-45	**-**	**-**			
Vulva (L) ^a, f^	-	**0.058-0.233^e^**	-	0-0.110^e^	-	0.047-0.094^e^	-	0.076-0.150^e^	-	0.090-0.150^e^	-	0.080-0.135^e^	**-**	0.107**-**0.149			
Eggs mature (L×W) ^c^	-	**43.27×21.52**	-	45-49×23-28	-	41-49×23-29	-	42-54×25-30	-	48-55×20-27	-	50-56×20-30	**-**	45**-**53×20**-**24			
Spicule (L) ^a^	**1.96-2.29**	-	1.25-1.77	-	1.04-1.36	-	1.19-1.32	-	2.20-2.60	-	2.20-2.53	-	1.48**-**1.49	**-**			
Spicule (W) ^c^	**8**-**12**	-	-	-	-	-	-	-	-	-	-	-	**-**	**-**			
ME/BL (%)	**2.41**	**1.78**	-	-	-	-	-	-	-	-	-	-	**-**	**-**			
TE/BL (%)	**39**	**24.30**	-	-	-	-	-	-	32.6-39.2	24.8-29.9	28.11-43.42	25.3-30.3	53	31**-**43			
# Specimen	**10**	**10**	6	6	4	9	6	6	10	10	8	8	6	6			
Reference	**In this study**		Sakaguchi et al., 2020						Garbin et al., 2021				Presswell & Bennett, 2022				

L: length; W: width; ME: muscular esophagus; TE: total esophagus; BL: body length; #: number.

a Measurements in milimeters.

b Esophageal-intestinal junction.

c Measurements in micrometers.

d Distance from front end.

e Junction of the intestinal esophagus to the vulva.

f Fragment.

g Moravec 2001 book.

h As recorded by the author.

i Buenos Aires, Laguna Chis-Chis.

j Buenos Aires, Laguna San Miguel del Monte.

Garbin et al. (2021) state that a complete morphological examination is necessary and must be accompanied by other approaches, including different molecular genetic analyses and evaluation of the geographic distribution of hosts of several species. In the present research, it was necessary to confirm the difference through molecular analysis, where the specimens were identified as the species *B*. *appendiculata*, since there was a genetic distance of 0.010 in relation to *B*. *spiculata* and 0.039 in relation to *B*. *obsignata*.

Sequencing and resequencing more species and large-scale comparative studies can also reveal and correct misidentifications or mislabeled datasets as per Smythe et al. (2019).That is the case with Tamaru et al. (2015) who carried out morphological and molecular characterizations of species of the Capillariidae family, considering the validity of the last classification of the family after the redefinition of Moravec (1982), based on male morphology as the most important morphological characteristic for separating the genera (Moravec, 1982; Gibbons, 2010). This paper provides the first report of the DNA 18S sequence of *B*. *appendiculata* parasitizing *P*. *brasilianus*.

In the present study the histopathological analysis of the sections of the cloaca revealed injuries in the mucosal layer, and intense inflammatory infiltrate due to the presence of nematodes, where plasmocytes, lymphocytes and some eosinophils were observed in the muscular layer of the mucosa. That was due to the high parasitic load of capillariids in the cloaca, differing from the types of cellularity present in the inflammatory infiltrate as described by Pinto et al. (2008) recorded *B*. *obsignata* in *Meleagris gallopavo* (Linnaeus), which caused thickening of the intestinal crypts and villi, together with a mild infiltrated mixed inflammatory picture, in the presence of mononuclear cells and heterophils. That also differs from Carvalho et al. (2021) who described histological changes caused by capillariids of the species *Eucoleus contortus* in the esophagus of the bird *Cairina moschata* (Linnaeus), in which the inflammatory infiltrate predominantly consisted of eosinophils.

## Conclusion

This is the first record of *B*. *appendiculata* parasitizing the cloaca of *P*. *brasilianus* from Marajó Island, State of Pará, Brazil, based on integrative taxonomy, using morphological, morphometric, and molecular data. The histopathological analysis of the lesions caused by this parasitism was reported.
